# The Effects of Asbestos Fibers on Human T Cells

**DOI:** 10.3390/ijms21196987

**Published:** 2020-09-23

**Authors:** Naoko Kumagai-Takei, Suni Lee, Bandaru Srinivas, Yurika Shimizu, Nagisa Sada, Kei Yoshitome, Tatsuo Ito, Yasumitsu Nishimura, Takemi Otsuki

**Affiliations:** 1 Department of Hygiene, Kawasaki Medical School, 577 Matsushima, Kurashiki, Okayama 701-0192, Japan; kumagai@med.kawasaki-m.ac.jp (N.K.-T.); slee@med.kawasaki-m.ac.jp (S.L.); srinivasgen08@gmail.com (B.S.); yurika.s0617@s.okayama-u.ac.jp (Y.S.); nagisada@okayama-u.ac.jp (N.S.); kei_y@med.kawasaki-m.ac.jp (K.Y.); tataito@med.kawasaki-m.ac.jp (T.I.); yas@med.kawasaki-m.ac.jp (Y.N.); 2 Department of Pathophysiology-Periodontal Science, Okayama University Graduate School of Medicine, Dentistry and Pharmaceutical Sciences, Okayama 700-8558, Japan; 3 Department of Biophysical Chemistry, Graduate School of Medicine, Dentistry and Pharmaceutical Sciences, Okayama University, Okayama 700-8530, Japan

**Keywords:** asbestos, T cell, cytotoxic T lymphocyte, CD4+ T cell, regulatory T cell, pleural plaque, malignant mesothelioma

## Abstract

Asbestos exposure causes malignant tumors such as lung cancer and malignant mesothelioma. The effects of asbestos fibers on immunocompetent cells, however, have not been well studied. Asbestos physically comprises a fibrous substance, which differs from silica particles which are a particulate substance, although chemically it is a mineral silicate. Since silicosis patients previously exposed to silica particles often suffer from lung and autoimmune diseases, it is clear that silica exposure impairs immune tolerance. Similarly, asbestos may alter the immune system in asbestos-exposed individuals. Given that malignant tumors can result following exposure to asbestos, the attenuation of anti-tumor immunity in cases of asbestos exposure is an important area of investigation. We observed the effect of asbestos fibers on T lymphocytes, such as CD8+ cytotoxic T lymphocytes (CTLs), CD4+ helper T (Th), and regulatory T (Treg) cells, and showed that anti-tumor immunity was attenuated, as demonstrated in a system that stimulates fresh cells isolated from peripheral blood in vitro and a system that is continuously exposed to a cell line. In this manuscript, we introduce the experiments and results of studies on CTLs, as well as Th and Treg cells, and discuss how future changes in immunocompetent cells induced by asbestos fibers can be clinically linked.

## 1. Introduction

Asbestos fibers are natural fibers that form part of the serpentine and amphibole groups. The former only includes one type of fiber called chrysotile (white asbestos) [1,2]. This forms a layered or sheet type of structure. This fiber displays a curved, curly, and wavy morphology. Additionally, the ends of the fiber bundles possess a splayed appearance. On the other hand, the amphibole group includes the fibers crocidolite (blue asbestos), amosite (brown asbestos), tremolite, anthophyllite, and actinolite. These display a linear form with rigid and firm features. Asbestos fibers are heat resistant, resistant to chemical corrosion, impermeable to electricity, heat, and sound, resistant to friction, and relatively inexpensive [3,4,5]. Consequently, they were used for many years in a wide variety of industries [3,4,5]. However, asbestos fibers are known to cause various health issues including various types of pneumoconiosis, such as asbestosis. Even though this is not a malignant disease, most patients develop life-threatening lung fibrosis with respiratory failure [6,7,8]. Additionally, asbestos can cause various benign diseases such as pleural plaque (PP), benign asbestos effusion, rounded atelectasis, and diffuse pleural thickening [6,7,8]. However, the most serious diseases caused by asbestos fibers are malignant diseases such as malignant mesothelioma (MM) and lung cancer [9,10,11].

Conventionally, a cell culture experimental system has been employed where alveolar epithelial or pleural mesothelial cells are transiently exposed to asbestos fibers in an effort to determine the biological effects of fibers, particularly on cellular and molecular events related to carcinogenesis. Crocidolite and amosite fibers contain a relatively large amount of iron. Additionally, chrysotile, which chemically comprises no iron, does contain some amphibole group contamination as a natural mineral, and the presence of iron is important in any case [12]. Some reports have shown that chrysotile can include a small amount of iron [13], as suggested by results obtained from experimental models of DNA damage due to the production of reactive oxygen species (ROS), and the enhancement of apoptosis via mitochondria [14,15,16,17]. Transient exposure to asbestos in animal or cell models, however, does not truly reflect the manner in which humans can be exposed to asbestos on a long-term basis. Although the inhalation of asbestos fibers by humans can be infrequent, the fibers are retained in the lungs, pleura, or lymph nodes [18]. Thus, lung epithelial, pleural mesothelial, and circulating immune cells can be in contact with these fibers in a chronic and relatively low-dose manner [19]. Taken together with results of experimental systems involving chronic exposure to asbestos at low concentrations, nuclear DNA damage accumulates, and even if apoptosis does not occur, it may occur somewhere. The acquisition of resistance to the induction of apoptosis is considered to be the carcinogenic mechanism invoked within target cells [12,13,14,15].

On the other hand, the effects of asbestos on immunocompetent cells have not been discussed so far. In chemical terms, asbestos may be categorized as a mineral salt of silica (SiO_2_) [1,2]. Silica is particulate in form while asbestos is fibrous. This difference is important in determining which type of pathological lesion that may occur. Examples include nodule formation in the middle to upper lung fields in silicosis versus honey-comb type fibrosis in the middle to lower lung fields in asbestosis [6,7,8,20]. Asbestos fibers comprise a complex crystal structure that includes silicate and other compounds. For example, the chemical formula of crocidolite and amosite are Na_2_Fe^II^_3_Fe^III^_2_Si_8_O_22_(OH)_2_ and Fe_7_Si_8_O_22_(OH)_2_, respectively [12]. However, in cases of silicosis, which can result following exposure to silica [21,22], complications of autoimmune disease [23,24] are well known in addition to complications of the lung [21,22]. There are many reports of systemic sclerosis [25,26] and anti-neutrophil cytoplasmic antibody (ANCA)-related vasculitis [27,28], including Caplan’s syndrome, which is known to be associated with rheumatoid arthritis in cases of coal miners with pneumoconiosis [29,30]. That is, it is assumed that exposure to silica will have some effect on immunocompetent cells, and we have also reported on some of these analyses. In physical terms, although there is a big difference between particulate and fibrous matter, there is a chemical similarity between silica and asbestos, and if silica affects immunocompetent cells, asbestos fibers might also have some effect. Since the most serious and problematic disease resulting from exposure to asbestos is development of a malignant tumor such as lung cancer or MM, the effect of asbestos on immune cells should be observed from the viewpoint of anti-tumor immunity.

In this review, we analyzed the results of our investigations on the effect of asbestos on T lymphocytes [19,31,32]. Our new findings regarding the role played by oxidative stress and transcription factors are also included in this review. Among these, the effects on CD8+ cytotoxic T Lymphocyte (CTL) and CD4+ helper T (Th) cells as well as the effects on regulatory T (Treg) cells will be summarized. Chronic activation in vitro of freshly isolated peripheral blood lymphocytes (PBLs) derived from healthy volunteers (HV) was employed. Additionally, human non-tumorous cell lines, EBT for CTL and MT-2 for Treg (see below for details), were subjected to chronic exposure to asbestos fibers at relatively low doses. Furthermore, the cellular and molecular alterations found in these experimental models were confirmed using PBLs derived from PP or MM patients that had previously been exposed to asbestos.

All findings suggested that anti-tumor immunity had been attenuated by gradual exposure of immune cells to asbestos. This slow attenuation over a period of 30 to 50 years may give rise to asbestos-related malignant tumors, especially given the diminished power of immune surveillance to counter cancer cells. This scenario is consistent with the occurrence of MM, which has a latency period of 30 to 40 years since initial exposure to fibers. The attenuation of anti-tumor immunity can be taken into account when immune checkpoint inhibitors are used for the treatment of MM and other diseases.

## 2. Effect of Asbestos Fibers on Various T Lymphocytes

### 2.1. Effects on CTLs

**Figure 1:**
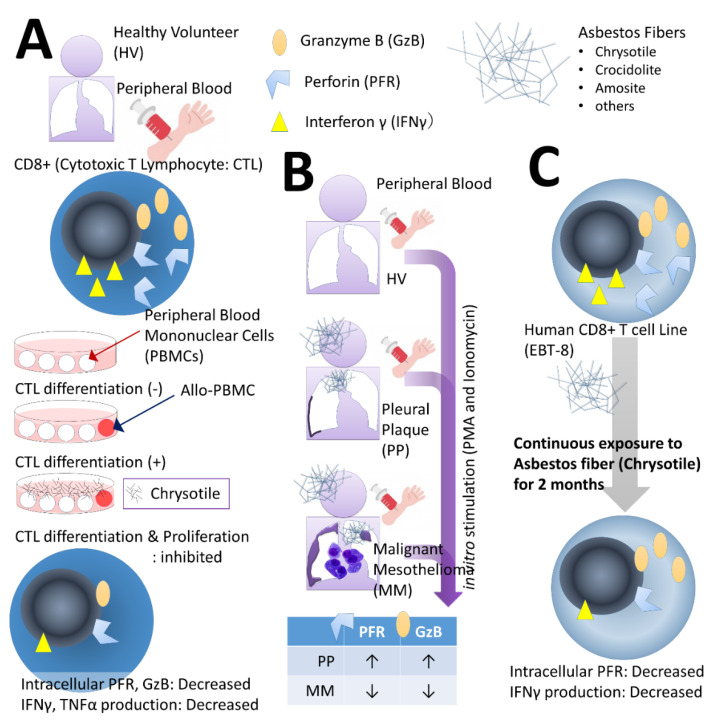
Observation of the effect of asbestos fibers on CD8+ cytotoxic T lymphocytes (CTLs). In the peripheral blood mononuclear cells (PBMCs) collected from (**A**) healthy volunteers (HVs), clonal expansion of CTLs was observed in the mixed lymphocyte reaction (MLR). When co-cultured with allogenic PBMCs, CD8+ cells differentiated into CTLs and proliferated, but when chrysotile and asbestos fibers were added, both differentiation and proliferation were suppressed. Furthermore, expression of granzyme B (GzB) and perforin (PFR), which execute the cell killing mechanism, and the production of interferon (IFN)-γ and tumor necrosis factor (TNF)-α, which are closely related cytokines, were also reduced. (**B**) With HV, CD8+ cells were isolated from the peripheral blood of patients with pleural plaque (PP) and malignant mesothelioma (MM), stimulated with phorbol 12-myristate 13-acetate (PMA) and ionomycin overnight, and expression levels of PFR and GzB in the chamber were observed. In the PP group, both were higher compared with HV and decreased in the MM group. (**C**) After the human CD8+ cell line ETV-8 was continuously exposed to chrysotile and asbestos fibers for 2 months, intracellular PFR decreased and IFN-γ production decreased.

Figure 1 shows a summary of the observations of the effect of asbestos fibers on CTLs. In these observations, three main studies were conducted.

The first comprises the experimental system referred to as the mixed lymphocyte reaction (MLR) using peripheral blood mononuclear cells (PBMCs) obtained from peripheral blood of HV depicted in Figure 1A [33,34]. PBMCs from HV were divided into three groups comprising (1) PBMCs only, (2) PBMCs cultured with allogenic PBMCs for the MLR, and (3) PBMCs cultured with allo-PBMCs and co-cultured with asbestos fibers (chrysotile). Chrysotile was used since it is the most abundantly used asbestos fiber and because marked differences between chrysotile and crocidolite were absent with regard to cellular and molecular changes observed in the MT-2 cell line subjected to chronic exposure (see below for details) [35]. When co-cultured with allogenic PBMCs, CD8+ lymphocytes differentiate and proliferate into CTLs (clonal expansion). However, co-culture with fibers suppressed differentiation and proliferation. Furthermore, intracellular expression levels of granzyme B (GzB), a molecule that executes the cell killing mechanism, and perforin (PFR) were also attenuated by co-culture with fibers. Moreover, the synthesis of interferon (IFN) γ and tumor necrosis factor (TNF) α, which are important cytokines for cell killing function, also decreased. These results suggest that exposure to asbestos fibers may impair tumor immunity in CTLs [33,34].

Secondly, in addition to HV, PBMCs were collected from the peripheral blood of patients with PP and MM, and CD8+ cells therein were stimulated in vitro with phorbol 12-myristate 13-acetate (PMA) and ionomycin. The expression level of GzB and PFR in cells was examined. As a result, both were higher in PP and lower in MM compared with those from HV (Figure 1B) [36].

The cause of PP is thought to be related to the insertion of shortened asbestos fibers into the tissues under the mesothelial layer, which then triggers fibrotic connective tissue reactions. Although the cause of MM has yet to be fully delineated, asbestos fibers protruding from the edge of alveoli stimulate mesothelial cells which causes chronic inflammation, DNA damage from ROS, and escape from apoptosis [3,4,5,6,7,8]. Any immunological differences may reasonably reflect pathophysiological differences. Of course, PP is not reflective of a precancerous state. Even though patients with PP had a history of asbestos exposure, the patients could not recall the circumstances of the exposure. At present, we do not possess any established tools such as the identification of biomarkers against MM [3,4,5,6,7,8], and the initial screening of individuals with a history of asbestos exposure is by way of a chest X-P in an effort to detect PP as a means of confirming exposure [3,4,5,6,7,8,37]. However, MM can occur in patients with PP as well as non-plaque individuals. If these two pathological conditions can be clearly divided using an immunological score (relative to HV), the benefit in screening asbestos-exposed individuals to detect PP or MM is apparent [35]. In any case, MM patients showed reduced anti-tumor immunity [36]. It is presently unclear which is the cause and which is the result—the reduction in anti-tumor immunity or the occurrence of mesothelioma—since cancerous cells may affect immune cells in a manner that supports the former’s growth and proliferation. However, our findings indicate that a long-term gradual reduction in the tumor surveillance system seems to allow for initial transformation of cancer-like cells caused by asbestos fibers. Further studies are required to shed light on this possible mechanism.

Furthermore, in order to observe the effects of continuous low-concentration exposure in detail, we used the human-derived CD8+ cell line EBT-8 (kindly provided by Professor Asada of Nara Medical University, [38]) [39]. Continuous exposure to chrysotile was performed for about two months, and changes in molecules related to anti-tumor immunity were observed (Figure 1C) [39]. As a result, there were no obvious changes in GzB, but PFR and IFN-γ production were attenuated. This result is also similar to the MLR result and indicates that asbestos exposure seems to influence CTLs in terms of diminishing their anti-tumor armamentarium. However, as revealed by examination of cases of PP and MM, the attenuation of anti-tumor activity appears to be complicated and may vary depending on factors hitherto unknown. In this context, assuming that the function of CTLs is regulated to suppress carcinogenesis, it may be possible to identify targets that can be utilized in chemoprevention efforts with exposed individuals by identifying the factors involved [39].

### 2.2. Effects of Th Cells

**Figure 2:**
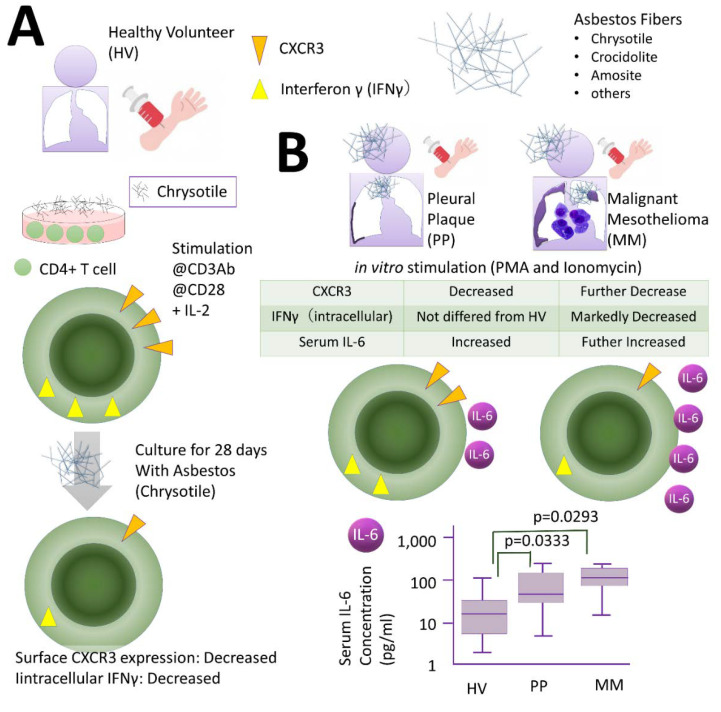
Effect of asbestos fibers on Th cells. (**A**) CD4+ T lymphocytes were isolated from HV peripheral blood and treated with interleukin (IL)-2 together with anti-CD3 and anti-CD28 antibodies for 4 weeks to induce clonal expansion. Expression levels of CXC chemokine receptor (CXCR) 3 on the cell membrane surface changed markedly. Intracellular IFN-γ expression levels were also reduced. (**B**) CD4+ cells were collected from the peripheral blood of individuals with PP or MM who were actually exposed in vivo, and these cells were stimulated overnight with PMA and ionomycin in an effort to observe changes in CXCR3 on the membrane surface and intracellular IFN-γ. CXCR3 levels diminished in cells derived from HV, and the PP and MM groups. However, although IFN-γ levels were lower in the MM group, they did not differ between HV and the PP group. When several representative cytokines related to HV, PP, and MM were measured, IL-6 levels in cells derived from the PP and MM groups were higher than those of HV. There was no statistically significant difference between the PP and MM groups, although the MM group tended to be slightly higher.

The effect of asbestos fibers on Th cells is shown in Figure 2.

Firstly, CD4+ T lymphocytes were isolated from HV peripheral blood and treated with interleukin (IL)-2 together with anti-CD3 antibody and anti-CD28 antibody for 4 weeks to induce clonal expansion. Exposure to chrysotile fibers resulted in a marked change in expression of CXC chemokine receptor (CXCR) 3 on the cell membrane surface. T cells expressing CXCR3 molecules are known to recruit T cells that attack cancer cells in the periphery, and CXCR3 is therefore an important molecule for anti-tumor immunity. Furthermore, intracellular IFN-γ expression levels were also reduced. Both results suggested a decrease in anti-tumor immunity (Figure 2A) [40].

Furthermore, CD4+ cells were collected from the peripheral blood of individuals with PP or MM who were actually exposed in vivo, and these cells were stimulated overnight with PMA and ionomycin in an effort to observe changes in CXCR3 on the membrane surface and intracellular IFN-γ. CXCR3 levels diminished in cells derived from HV, and the PP and MM groups. However, although IFN-γ levels were lower in the MM group, they did not differ between HV and the PP group. This is similar to the result observed with CTLs, and the peculiarity of PP was suggested (Figure 2B) [40].

When several representative cytokines related to HV, PP, and MM were measured, IL-6 levels in cells derived from the PP and MM groups were higher than those of HV (Figure 2B). There was no statistically significant difference between the PP and MM groups, although the MM group tended to be slightly higher. An interpretation of this result is rather difficult and may require a discussion of T cell polarity. For example, when both transforming growth factor (TGF) beta and IL-6 are present, Treg cells are present in the T cell population whose polarity is induced by the environment. If Treg cell function or numbers increase, then anti-tumor immunity as a whole moves in the direction of attenuation. If asbestos exposure is induced, as is often the case with carcinogenesis, this is also a phenomenon that must be fully considered [40].

### 2.3. Effects of Treg Cells

Since Treg cells suppress T cells that attack cancer cells, if Treg cell function or numbers increase, then anti-tumor immunity is attenuated. The effect of asbestos fibers on Treg cells was examined using cell lines. It was reported that an MT-2 cell line immortalized with human T cell leukemia/lymphoma virus (HTLV)-1 possesses Treg function [41].

**Figure 3:**
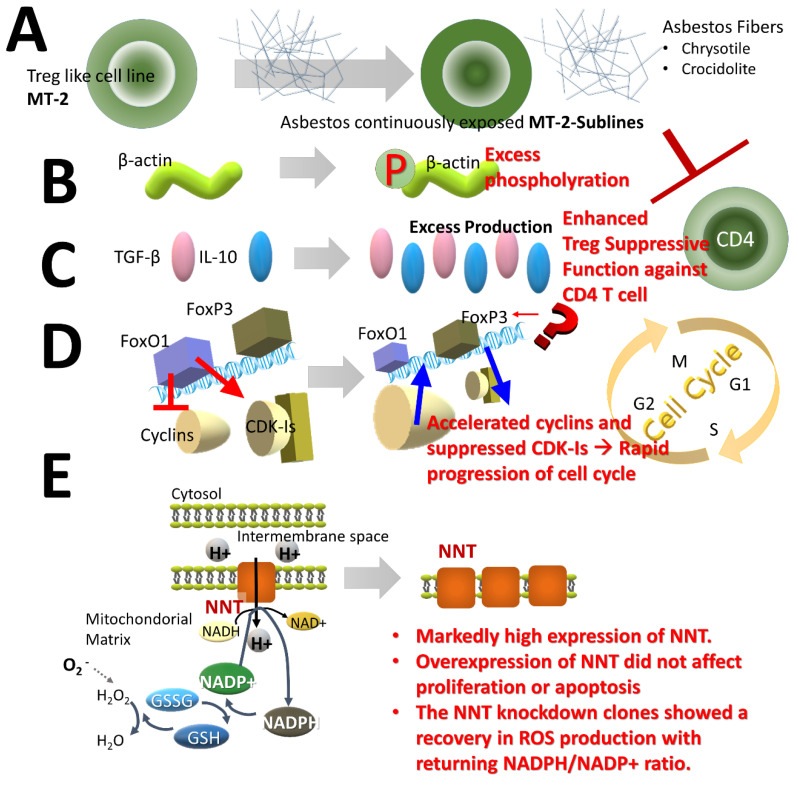
Summary of the effect of asbestos on Treg cells using the Treg-like cell line MT-2. (**A**) The MT-2 line resists apoptosis after continuous exposure for as long as one year at a concentration of asbestos fibers (chrysotile or crocidolite) that induces apoptosis in less than half the cell population following transient exposure. Sublines that acquired resistance to apoptosis were established. (**B**) In terms of protein expression, excessive phosphorylation of β-actin occurred in sublines. (**C**) Cytokines cause overproduction of IL-10 and transforming growth factor (TGF)-β, which are soluble factors required for expression of typical Treg cell function, together with cell–cell contact, and functional enhancement was shown. (**D**) Among the transcription factors, forkhead box protein O1 (FoxO1) was significantly attenuated, and negative regulation of cyclins with positive regulation of cyclin-dependent kinase-inhibitors (CDK-Is) accelerated the cell cycle. Asbestos-exposed Treg cells were over-functional and abundant. Expression of FoxP3, an important Treg cell transcription factor, was slightly attenuated in spite of hyperactivity, although no other significant changes such as methylation were observed. (**E**) Furthermore, nicotinamide nucleotide transhydrogenase (NNT) was highly expressed due to the relationship between oxidative stress and oxidative phosphorylation. This may act to protect asbestos-exposed Treg cells from oxidative stress and may also play a role in their escape from cell death.

Transient exposure of asbestos fibers to MT-2 cells causes phosphorylation of p38 and JUN, which are pro-apoptotic molecules of ROS production and the mitogen-activated protein kinase (MAPK) pathway, thereby inducing apoptosis via activation of the mitochondrial pathway [42]. The concentration of chrysotile or crocidolite fibers at which transient exposure induced apoptosis in less than half the cell population was determined. Continuous long-term exposure of sublines (six sublines for chrysotile and three sublines for crocidolite were independently isolated) was then performed (Figure 3A) [35], and the characteristics of these sublines were compared with the parent MT-2 original line. A comparison of protein expression suggested that hyper-phosphorylation of β-actin occurred in the sublines [43]. Additionally, enhanced binding of cytoskeletal proteins such as vimentin and tubulin to asbestos fibers was also observed. These results suggest that asbestos fibers themselves are not taken up into cells (due to their length and physical properties) [43], so that changes in molecules related to the cytoskeleton were induced (Figure 3B) [43]. A number of reports showed that asbestos fibers can pierce the cell membrane and reach the cell interior [44,45]. Consequently, recurrent encounters of fibers with the cell membrane may occur together with intracellular damage, and both events can alter the cell features of immune cells.

In addition, it was noted that expression and production of cytokines IL-10 and TGFβ were markedly increased (Figure 3C) [35,41,46]. These molecules represent two typical cytokines that are important when Treg cells exert their function. We examined Treg cell function in sublines and the original cell line. In addition to cell proliferation of Th cells being suppressed by cell-to-cell contact, it was found that these two cytokines effectively act to suppress function [47]. This suggests that Treg cells exposed to asbestos possess hyperactivity, which may result in diminished anti-tumor immunity.

Furthermore, expression of transcription factor forkhead box protein O1 (FoxO1) was significantly reduced [48]. FoxO1 regulates many genes, including genes involved in cell cycle regulation. By controlling cyclins, which accelerate the cell cycle, in a negative manner and cyclin-dependent kinase-inhibitors (CDK-Is) in a positive manner, regulation can act to brake or curtail the entire cell cycle. However, it was found that decreased expression of cyclins and decreased expression of CDK-Is caused acceleration of the cell cycle in sublines [49]. This was also regulated by FoxO1, as revealed by overexpression by transfection into sublines or silencing in the original MT-2 line. This suggests that Treg cells exposed to asbestos tend to increase in number as a result of the hyperactivity described above and by acceleration of the cell cycle. Again, this facilitates a move towards the attenuation of anti-tumor immunity (Figure 3D). Expression of forkhead box P3 (FoxP3), which is a master gene of Treg cells, showed a slight decrease in spite of Treg cell hyperactivity. We then examined methylation changes and the expression of other transcription factors related to Treg cells, but no clear difference was found between sublines and the original line not treated with asbestos fibers [50]. It is therefore considered that the changes induced in Treg cells following asbestos exposure relate to cell hyperactivity and growth promotion, although the molecular mechanisms involved remain to be delineated. However, increased Treg cell function and number following asbestos exposure may be important when considering molecular targets and immune checkpoints in the treatment of asbestos-induced malignancies among asbestos-exposed individuals.

Finally, although not directly related to Treg cell function, interesting results were obtained in experiments using this MT-2 cell line. As mentioned above, ROS regeneration is a typical effect when cells are exposed to asbestos fibers [6,7,8]. Thus, the expression pattern of enzymes related to anti-oxidative stress and redox reactions may be altered in continuously exposed sublines since sublines acquired resistance against asbestos-induced apoptosis [41]. If sublines can effectively render harmless ROS produced following contact with asbestos, certain alterations in the oxidative phosphorylation (OXPHOS) complex may occur in sublines. The complex comprises five multimeric enzymes located on the mitochondrial membrane [51,52]. Complexes I to V are important for oxidative phosphorylation. Additionally, nicotinamide nucleotide transhydrogenase (NNT) also transports H+ from the intermembrane space to the inside. When we examined protein and mRNA expression of molecules comprising OXPHOS, we found that NNT levels were extremely high in sublines (Figure 3E) [53]. This was unrelated to subline proliferation or changes in apoptosis. Interestingly, however, ROS production remained suppressed in an NNT-highly expressed subline when examining the ratio of reduced nicotinamide adenine dinucleotide phosphate (NADPH) to oxidized NADP+ (NADPH/NADP+). Furthermore, changes in the ratio of NADPH to oxidized NADP+ (NADPH/NADP+) also acted to relieve oxidative stress due to asbestos (Figure 3E) [5].

It is still unclear how the preceding remarks concerning NNT relate to Treg cell features and other phenomena. However, if acting to relieve oxidative stress, these events may have altered Treg cells to escape apoptosis following asbestos exposure and DNA damage, which in turn may have an impact on long-term survival and other processes. This indicates that at the very least these events act to prevent the disappearance of Treg cells, thereby attenuating anti-tumor immunity.

## 3. Discussion

We reviewed our research results on the effects of asbestos on immunocompetent cells. Long-term continuous exposure to asbestos in all T lymphocytes of CTL, Th, and Treg cells showed diminished anti-tumor surface immunity [54,55]. As an aside, we also conducted a similar study on natural killer (NK) cells, which are not T lymphocytes, but are important immunological cells in anti-tumor immunity, and asbestos exposure caused functional activation of cell membranes. It was shown that cell killing function is attenuated through decreased expression of the receptor NKp46. Similarly, through MAPK, signal transduction was attenuated, and PFR secretion was suppressed. Furthermore, the expression level of NKp46 was lower in PP than in MM.

All of these findings suggested that continued exposure of T lymphocytes to asbestos fibers impaired anti-tumor immunity. However, Th17 cells have yet to be examined. In this regard, some groups are conducting research using asbestos-exposed individuals or animal models of autoimmune diseases, and it is expected that the results will be forthcoming. Th17 cells seem to be more strongly involved in the breakdown of self-tolerance rather than anti-tumor immunity [56,57,58]. This area requires extensive further investigation.

This review focused on the effects of asbestos fibers on immune cells with particular emphasis on circulating immune T lymphocytes from the viewpoint of general anti-tumor immunity. Inhaled asbestos fibers remain at the lung, pleura, and lymph nodes. As a result, asbestos-induced cancers can occur at the sites where fibers remain such as the lung and pleura. Therefore, the immune microenvironment should be taken into account with any consideration of the immunological effects of asbestos fibers. Chu et al. recently reported on unsuccessful trials of immune checkpoint inhibitors in the treatment of MM [59]. They discussed various immune cells surrounding MM tumor cells such as T cells, tumor-associated (M2) macrophages, and polymorphonuclear myeloid-derived suppressor cells (PMN-MDSC), as well as cancer-associated fibroblasts. Although their approach differed from ours (general immune status versus microenvironment of tumor), there are several features common to both approaches, such as increasing CD4+FOXP3+ and increased TGFβ-Treg cells with ROS competition. Additionally, they suggested that these changes may be partially caused by hypoxia and hypoglycemia in the tumor-surrounding microenvironment, and that the immune suppressive microenvironment in mesothelioma is likely to cause failure of immune checkpoint inhibitor treatments. They showed how circulating T cells exposed to asbestos alter their cellular and molecular characteristics by hypoxia as well as hypoglycemia. These mechanisms may play a role in determining the outcome when circulating T cells already affected by asbestos fibers proceed to the tumor surrounding lesion.

Huaux recently reviewed immunosuppression in diseases induced by particles, including micro- and nanoparticles [60]. Of course, that review included the effects of silica particles and discussed the cellular and molecular effects of various particles on inflammation including fibrosis, cancer, infection, and autoimmunity. Various conditions associated with inflammation may proceed to immunosuppression. This immunosuppression was related to such events as fibrosis, cancer, and infection. The review showed the importance of cytokines and various immune cells such as M2 macrophages, MDSCs, Treg cells, and B cells. Important cytokines with respect to immunosuppression are IL-10 and TGFβ. As far as inflammation is concerned, ROS and reactive nitrogen species (RNS) are considered to be important together with cytokines such as IL-17, IFNs, TNFα, IL-1β and IL-1α. Although very small nano-particles may penetrate the alveoli wall to various organs in the body, these inflammatory and immunosuppressive scenarios may be considered to occur at the site where inhaled particles remain, such as pulmonary, pleural, and local lymph nodes. We also investigated the effects of silica particles on human immune cells and found an imbalance of responder Th and Treg cells, which corresponded with studies of various autoimmune diseases [61,62,63].

However, those studies focused on circulating immune cells instead of local cells, since interaction between silica particles and circulating immune cells may be chronic and recurring at sites where particles remain in the body. This scenario resembles that of asbestos fibers that remain in the body as mentioned above. Thus, a consideration should be made of the effects of fibrous and particulate environmental substances on human immune cells at localized sites such as the lung, pleura and local lymph nodes, as well as encounters of these substances with the general immune system circulating in the body.

Comar et al. reported that the chemokines CXCL5 (IP-10) and CCL5 (RANTES) were associated with the degree of severity of asbestos-related diseases such as MM [64]. Additionally, RANTES, IP-10, and vascular endothelial growth factor (VEGF) were detected in mesothelioma biopsy specimens [64]. These results are very interesting when considering the immune state in MM. For IL-10, our continuous exposed cell line model using MT-2 cells showed lower levels of IP-10 in exposed sublines compared with the original non-exposed line [65]. On the other hand, we also checked the plasma levels of IP-10 among HV, PP, and MM. PP and MM showed higher IP-10 concentrations compared with HV. Additionally, in the MM group, CXCR3 expression in CT4+ cells and serum IP-10 showed a significant reverse correlation (*p* < 0.05). However, in PP, this significance disappeared and only showed a statistical “tendency” (0.1 > *p* > 0.05). Additionally, in HV, there was no correlation. Unfortunately, we have yet to measure RANTES, but IP-10 analyses indicated that asbestos fibers affect both immune cells and tumor cells. Tumor cells may act by editing or altering immune cells. Asbestos reduced IP-10 production in T cells, which may have an impact on mesothelioma cells since IP-10 is the ligand for CXCR3. Thus, IP-10 may be secreted by tumor cells in increasing amounts since expression of CXCR3 by T cells is reduced with asbestos exposure [40,65]. RANTES is present in platelet alpha-granules and is released in the supernatant with platelet stimulation. It exhibits strong migratory activity against eosinophils in cooperation with IL-2, IL-5, and IFN-γ. Furthermore, it possesses activity against memory T cells and monocytes, is involved in eosinophil activation, and affects degranulation and adhesion. It has been pointed out that RANTES is in some way associated with allergic inflammation because of its selective migration activity on memory T cells [66,67]. Additionally, an increase in platelet derived growth factor (PDGF) in MM has been reported [68,69]. Thus, as with IP-10, a consideration should be given to the possible involvement of RANTES in crosstalk between MM, asbestos, immune cells, and mesothelioma cells.

## 4. Conclusions

We observed the effect of asbestos fibers on T lymphocytes and found that anti-tumor immunity is attenuated in CD8+ cytotoxic T lymphocytes (CTLs), CD4+ helper T (Th), and regulatory T (Treg) cells. This was demonstrated using a system that stimulates fresh cells isolated from peripheral blood in vitro and a system that is continuously exposed to a cell line. In this manuscript, we introduced the experiments and results of studies on CTL, Th, and Treg cells, and discussed how the changes in immunocompetent cells induced by asbestos fibers could be clinically linked to disease outcome. It is hoped that these findings will lead to the development of effective diagnostic and chemoprevention strategies.

## Abbreviations

PP	pleural plaque
MM	malignant mesothelioma
ROS	reactive oxygen species
ANCA	anti-neutrophil cytoplasmic antibody
CTL	cytotoxic T Lymphocyte
Th	CD4+ helper T cell
Treg	regulatory T cell
HV	healthy volunteers
PBL	Peripheral blood lymphocyte
MLR	mixed lymphocyte reaction
PBMC	peripheral blood mononuclear cell
GzB	granzyme B
PFR	perforin
IFN	interferon
TNF	tumor necrosis factor
PMA	phorbol 12-myristate 13-acetate
IL	interleukin
CXCR	CXC chemokine receptor
TGF	transforming growth factor
HTLV	human T cell leukemia/lymphoma virus
FoxO1	forkhead box protein O1
CDK-Is	cyclin-dependent kinase-inhibitors
FoxP3	forkhead box P3
NNT	nicotinamide nucleotide transhydrogenase
NADPH	reduced nicotinamide adenine dinucleotide phosphate
NK	natural killer
